# 

**Published:** 1866-12

**Authors:** 


### CHICAGO

### MEDICAL JOURNAL.

Vol. XXIII. DECEMBER, 1866. No. 12.

CHICAGO:

JAMES BARNET, PRINTER, 191 LAKE STREET.

